# Preprocessing Ground-Based Visible/Near Infrared Imaging Spectroscopy Data Affected by Smile Effects

**DOI:** 10.3390/s19071543

**Published:** 2019-03-30

**Authors:** Henning Buddenbaum, Michael S. Watt, Rebecca C. Scholten, Joachim Hill

**Affiliations:** 1 Environmental Remote Sensing and Geoinformatics, Trier University, 54286 Trier, Germany; r.c.scholten@vu.nl (R.C.S.); hillj@uni-trier.de (J.H.); 2 Scion, 10 Kyle Street, Christchurch 8011, New Zealand; Michael.Watt@scionresearch.com; 3 Faculty of Earth and Life Sciences, Vrije Universiteit Amsterdam, 1081 HV Amsterdam, The Netherlands

**Keywords:** Specim FX10, field imaging spectroscopy, preprocessing, forestry, herbicide, invasive species

## Abstract

A data set of very high-resolution visible/near infrared hyperspectral images of young *Pinus contorta* trees was recorded to study the effects of herbicides on this invasive species. The camera was fixed on a frame while the potted trees were moved underneath on a conveyor belt. To account for changing illumination conditions, a white reference bar was included at the edge of each image line. Conventional preprocessing of the images, i.e., dividing measured values by values from the white reference bar in the same image line, failed and resulted in bad quality spectra with oscillation patterns that are most likely due to wavelength shifts across the sensor’s field of view (smile effect). An additional hyperspectral data set of a Spectralon white reference panel could be used to characterize and correct the oscillations introduced by the division, resulting in a high quality spectra that document the effects of herbicides on the reflectance characteristics of coniferous trees. While the spectra of untreated trees remained constant over time, there were clear temporal changes in the spectra of trees treated with both herbicides. One herbicide worked within days, the other one within weeks. Ground-based imaging spectroscopy with meaningful preprocessing proved to be an appropriate tool for monitoring the effects of herbicides on potted plants.

## 1. Introduction

To facilitate timber production in New Zealand, several coniferous tree species have been introduced from abroad. When they reproduce freely outside of plantations, they are considered invasive in their new environment [1,2]. In recent years, it has become evident that exotic trees are becoming invasive in many environments [3], and reports of their spread and adverse impacts have been reported from many regions [4]. The same evolutionary traits that make the introduced trees excellent timber species mean that they can often out-compete indigenous vegetation outside of their natural range [5]. Several conifer species including *Pinus contorta* (Dougl.), *P. mugo* Turra, *P. nigra* Arnold and *Pseudotsuga menziesii* (Mirb.) Franco have been spreading from commercial plantations, farm shelterbelts and woodlots for over 100 years into indigenous grasslands, shrublands, and even forested areas. 

The area affected by invasive exotic conifers is thought to cover approximately 2 million ha and is believed to be increasing at a rate of 6% annually [6,7]. The level of infestation has resulted in an economic and ecological cost that is increasingly deemed to be unacceptable by New Zealand society [8]. The elimination of invasive exotic conifer over large areas with difficult terrain is partly reliant on aerial herbicide application. Detection of the impacts of herbicides on conifers would be of considerable use for determining the efficacy of applied herbicides so that these can be optimally used for control. The use of hyperspectral imagery could provide a useful means of pre-visual detection as this imagery allows a detailed assessment of the wavelengths that are most closely correlated with physiological change and needle necrosis [9,10,11].

In order to understand the effect of herbicide application on spectral properties of lodgepole pine (*Pinus contorta* (Douglas)) trees, a controlled experiment with potted wilding trees was conducted. Two-thirds of the trees were treated with two different herbicides and the remaining trees formed the untreated control group. Close-range hyperspectral images of the trees were captured using a visible/near infrared (VNIR) camera fixed on a frame about 2 m above ground and a conveyor belt that moved the trees under the camera. Six captures were recorded over a five-week period (Supplementary Materials, Figures S1–S6).

Hyperspectral imaging, also known as imaging spectroscopy, has been successfully employed in many scientific fields from several platforms like satellite [12], airplane [13], field setups [14] and in the laboratory [15], e.g., for the determination of biochemical foliar traits of tropical trees [16], for the classification of tree species and age classes [17], or to map soil organic carbon [18]. Yao et al. [19] used a laboratory hyperspectral camera to detect herbicide damage in soybean plants before it could be detected visually. Suarez et al. [20] used hyperspectral data to detect herbicide drift in cotton fields. Zhao et al. [21] inverted a physically-based reflectance model on spectroscopic leaf measurements to detect herbicide crop injury. Recent reviews on hyperspectral remote sensing can be found in [22,23,24,25].

The sensors record either uncalibrated digital numbers (DNs) or, if they are calibrated, radiance [26]. These are dependent on illumination, so the researchers are usually rather interested in reflectance, an inherent trait of the material recorded. Reflectance is the ratio of radiant exitance with irradiance. The ratio of the radiant flux reflected by a surface to that reflected into the same geometry by an ideal and diffuse (Lambertian) standard surface is called reflectance factor [26]; this can be measured with a downward-facing sensor and a white reference panel.

Many imaging spectrometers suffer from shifts of the wavelength position across the field of view [27,28,29,30]. This is called the “smile effect” because a plot of wavelength position against the sensor column is often shaped like a smiling mouth. The intensity of the smile effect varies with the wavelength and with the position of the pixel within the field of view. Although the smile effect is usually quite small in the raw data, it can have serious consequences on the reflectance spectra since the DN or radiance spectra are not smooth, due to narrow atmospheric absorption bands.

One advantage of field or laboratory imaging spectroscopy over airborne or space-borne data is that the transformation from DN or radiance to reflectance (atmospheric correction) can be much simpler, especially if a reference target is recorded within the images [31,32,33,34,35]. When each image column also contains pixels of the reference target, the transformation from radiance to reflectance factors can be calculated for each column separately and the wavelength shifts across the field of view do not create a problem [34,36]. In the case of quickly changing illumination conditions, it may be more advisable to include a white reference in each line instead of each column of the image. If the camera employed suffers from shifts of the wavelength position across the field of view (smile effect) [27,28], the conversion from radiance to reflectance factors, through the division of measured values by the corresponding values of the white reference in the same image line, may fail and result in bad quality spectra. Here, we show that an additional capture of a white reference panel that covers the whole field of view of the camera can be used to characterize and correct these effects.

The objective of this research was to describe a method to robustly derive usable reflectance spectra from field imaging spectroscopy data affected by the smile effect. Within the materials and methods (Section 2), the herbicide experiment and the hyperspectral measurements are described. Then, the data analysis methods are presented, followed by the results (Section 3). Some short conclusions are drawn in Section 4.

## 2. Materials and Methods

### 2.1. Preparation of the Herbicide Experiment

For the herbicide experiment, 39 *Pinus contorta* plants that did not show any signs of previous herbicide treatment, fungi or other infections were collected from the field in the central North Island of New Zealand between February and March 2017. Tree age, as estimated from the number of whorls, averaged four years and ranged from three to six years. Following collection, plants were placed in a greenhouse to acclimatise for the nine months preceding the trial. Plants were fertilised with phosphorus and nitrogen and irrigated as required over the duration of the experiment. At the start of the experiment tree height, root collar diameter and canopy width, respectively, averaged 157 cm (range 110–205 cm), 3.34 cm (range 2.17–5.10 cm) and 74.3 cm (range 48–135 cm).

Stratified random sampling based on the total tree height was used to allocate the 39 trees to the three treatments that included an untreated control and two herbicide treatments. The two herbicides applied included the systemic pyridine herbicide, triclopyr butoxyethyl ester (Grazon, 600 g·L^−1^ triclopyr, Dow AgroSciences Ltd., New Plymouth, New Zealand) and the contact herbicide diquat dibromide monohydrate (Reglone, 200 g L^−1^ diquat dibromide, Syngenta, Australia).

On 20 March 2018, the two herbicide treatments were applied using a calibrated boom sprayer, fitted with Turbo Teejet nozzles (TT8003; Spraying Systems Co. Wheaton, Illinois, USA) at a pressure of 2.6 kPa, and at a height of 2 m above the seedling canopy. These nozzles produced a spray characterised by droplets with a volume mean diameter (VMD) ranging from 350–500 µm. After spraying, the trees were left to dry before being shifted back to the greenhouse on 21 March 2018, when the first imaging took place.

### 2.2. Hyperspectral Imaging

Hyperspectral measurements were conducted weekly before the treatment (capture 0) until five weeks after treatment (capture 5). A spectrograph-based Specim FX10 camera (Spectral Imaging Ltd., Oulu, Finland) was used to acquire the hyperspectral imagery. This instrument is a push-broom camera that captures 448 bands with wavelengths ranging from 400 to 1000 nm with a spectral sampling distance of 1.3 nm and a full width half maximum (FWHM) of 5.5 nm. The spatial sampling comprises 1024 pixels (columns) within a field of view of 38°. The camera records one line at a time (scanner principle), and images are formed from the single lines through movement of either the camera or the target. The image acquisition was managed using the provided Lumo Recorder software interface. The camera is not calibrated, so it records DNs.

The camera was mounted on a tower-like construction about 2 m above ground, and a conveyor belt was used to move the plants through the field of view. The speed of the conveyor belt was adapted to fit the frame rate of the camera, which in turn was dependent on the exposure time, which had to be adjusted to the current illumination conditions. During the trial, the conveyor belt speed was kept constant and the frame rate was only adjusted to match the exposure time. A diffuse narrow white reference reflectance bar was attached at the edge of the field of view so that it was visible in every frame allowing changing illumination to be accounted for during postprocessing.

### 2.3. Preliminary Smile Analysis

A preliminary smile analysis was conducted using an image of a white reference panel which was illuminated by sunlight and covered the camera’s complete field of view.

Eight wavelength regions with local minima in the signal due to atmospheric absorption or solar Fraunhofer lines were selected to characterize wavelength shifts. In each region, a second order polynomial was fitted to the data and the position of the minimum was found, separately for each of the 1024 sensor columns. If these minima were dependent on the sensor column, a wavelength shift was deemed to be present in the data.

### 2.4. Data Processing

To transform the measured digital numbers (DN) values to reflectance factors (R), they have to be divided by the respective white reference DN (WR) at wavelength λ [26]. In the case of the FX10 camera, the dark current has to be measured separately and subtracted from the signal [19,37]. For this purpose, a separate image with 100 lines is recorded while the camera shutter is closed. The mean of the 100 lines is calculated, resulting in a matrix of 1024 columns and 448 bands which can be subtracted from each image line. The resulting relative reflectance factor can be transformed into an absolute reflectance factor by multiplying the result with the reflectance of the white reference $R_{\lambda }^{Ref}$ [38] (Equation (1)):

(1) $$R_{\lambda }=R_{\lambda }^{Ref}\cdot \;\left(\frac{DN_{\lambda }-dark_{\lambda }}{WR_{\lambda }-dark_{\lambda }}\right).$$

Because of changing illumination conditions, each image line was corrected separately. As a first step, the pixels belonging to the white reference were identified; these were usually located around column 1000 of the 1024 image columns. The mean value of the five brightest pixels over the whole range of values was taken as WR in Equation (1).

**Figure 1:**
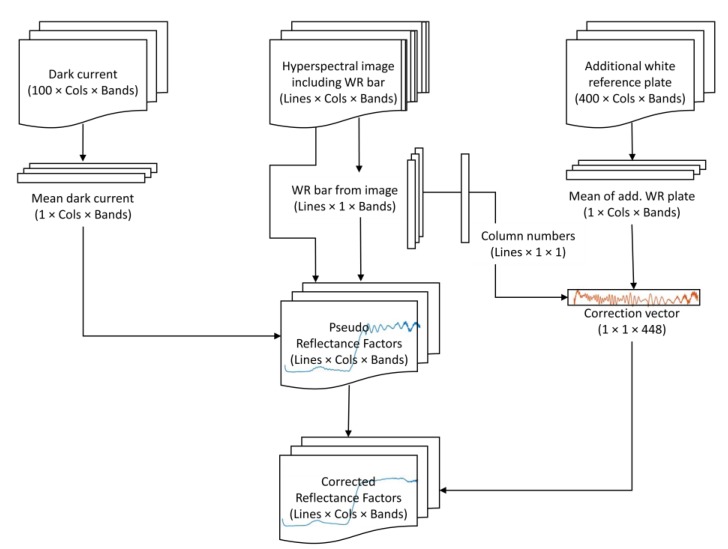
Data processing workflow: The mean dark current and white reference bar pixels of each line of the hyperspectral image are used to calculate pseudo reflectance factors. An additional hyperspectral data set of a white reference plate is transformed using the white reference bar pixel positions to create a correction vector which can be used to correct the pseudo reflectance data set. The size of each matrix is given. The camera employed in this study offers 1024 columns (cols) and 448 bands. The number of lines in the image and the image of the white reference plate are arbitrary.

This straightforward data processing scheme resulted in bad quality spectra that contained a wave pattern (pseudo reflectance). A simple solution to this problem was found when the white reference plate image that fills the camera’s whole field of view was treated the same way. After dark current subtraction, the white reference image was divided by its own mean values of the columns covered by the white reference bar in the original image. This results in a line with a mean value close to one that contains the same wave pattern. Dividing $R_{\lambda }$ by this line results in corrected reflectance factors. A flowchart of the processing scheme is shown in Figure 1.

In a further step, the images were filtered using three criteria: Pixels with normalized difference vegetation index (NDVI) values greater than 0.4, near infrared reflectance (800 nm) greater than 0.18 and blue reflectance (460 nm) less than 0.1 were accepted as vegetation pixels, the other pixels were masked out. Mean spectra of the trees were calculated as mean values of all vegetation pixels.

## 3. Results and Discussion

### 3.1. Analysis of Smile Effects

**Figure 2:**
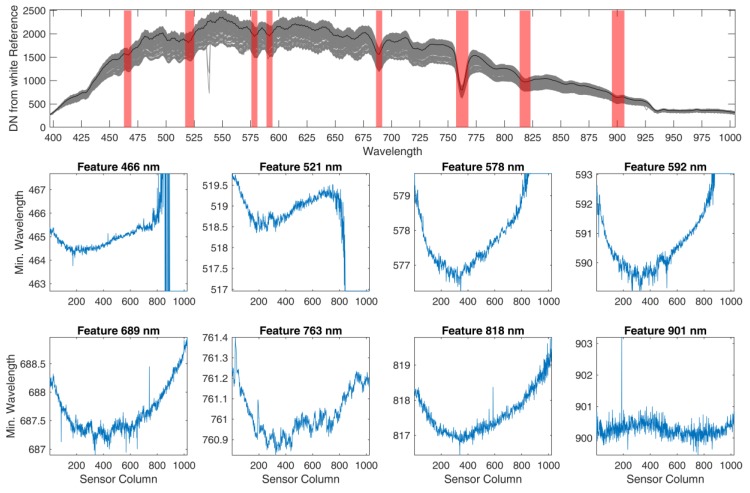
Analysis of sensor column dependent wavelength shifts. Top panel shows DNs of a measurement of sunlight reflected from a white reference panel (mean value in black and range given in gray). Eight wavelength positions with local minima are marked in red. The lower panels show the wavelength position of each minimum depending on the sensor column.

Results from the preliminary analysis of wavelength shifts are shown in Figure 2. For most absorption features examined, a smile shape displaying a clear minimum is evident. Due to the high number of local minima in the measured solar spectrum, the wavelength ranges of the selected features had to be quite narrow. For the first four features this leads to invalid values for column numbers greater than about 800, since there the minimum wavelength is located outside the defined wavelength range of the feature. The magnitude of the wavelength shifts is quite small, usually in the range of 1–2 nm, which is in accordance with the manufacturer’s calibration report. Still, this confirms that a treatment of smile effects may be necessary.

### 3.2. Calculation of Reflectance Spectra

**Figure 3:**
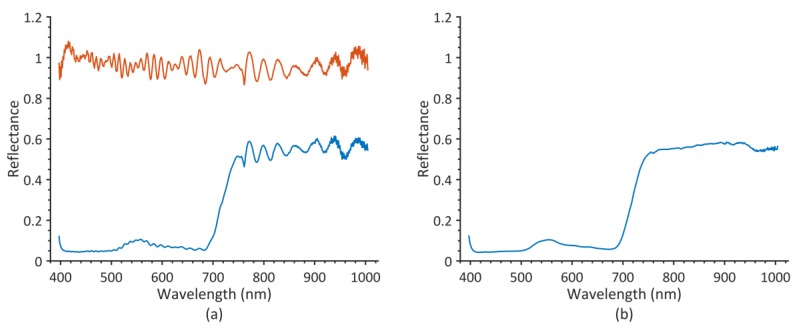
Calculation of reflectance spectra; (**a**) blue line shows the mean spectrum of a tree that results from division by white reference spectrum, red line shows the correction spectrum resulting from “correcting” an image of a white reference panel the same way; (**b**) corrected reflectance spectrum.

**Figure 4:**
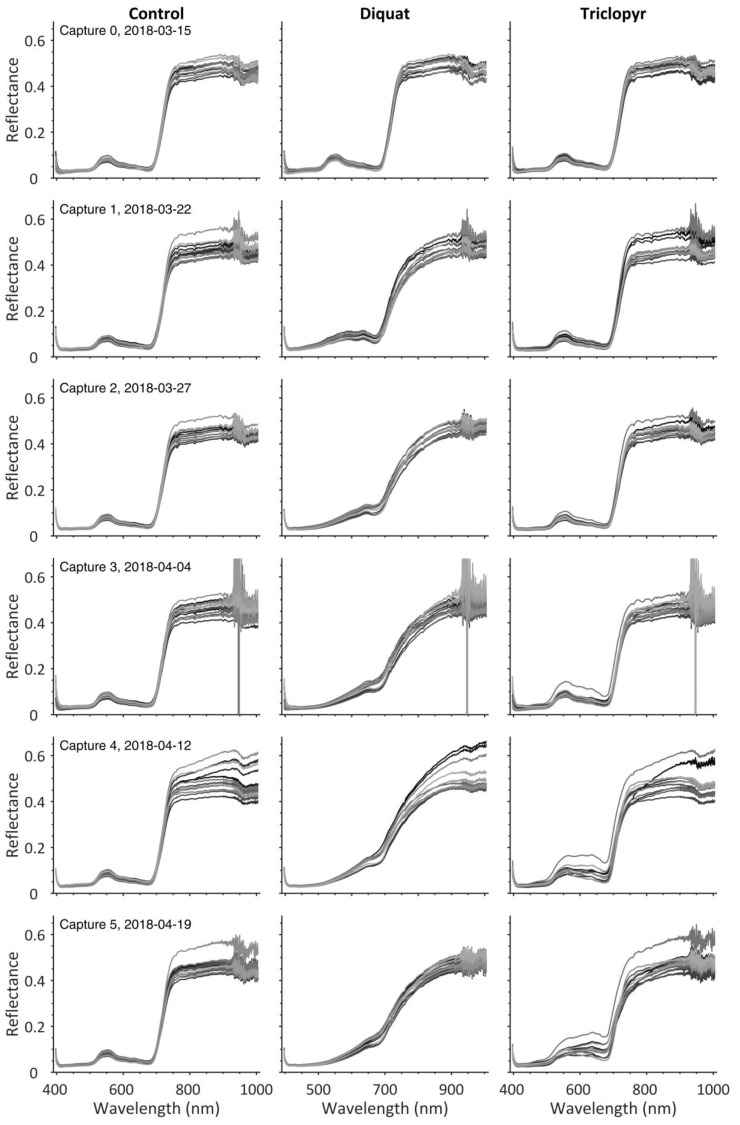
Corrected spectra of all trees. The left column contains the control group, the middle and right columns show the spectra of trees treated with herbicides.

Figure 3 shows the mean reflectance spectrum of the first capture of the first pot before and after correction of the wave pattern (mean of 53,700 pixels). The blue line in Figure 3a represents the ratio of vegetation pixels and the corresponding white reference bar pixels from the edge of the image. The red line is the ratio of white reference plate and pixels from the same edge position of the white reference plate image. This wave pattern mostly consists of an oscillation with oscillating amplitudes, with oscillation wavelengths increasing with spectral wavelength. Figure 3b shows the corrected spectrum, the result of the ratio of the two aforementioned spectra. Only very small oscillations are left in the spectrum. Corrected spectra of all trees are shown in Figure 4. A striking difference between spectra from different capture days is the varying amount of noise in the spectral region of 900 to 1000 nm. This can likely be explained by changing amounts of atmospheric water vapor between recording dates [39]. The quality of the spectra is high enough for further analysis of plant health and herbicide effects.

### 3.3. Hyperspectral Images

**Figure 5:**
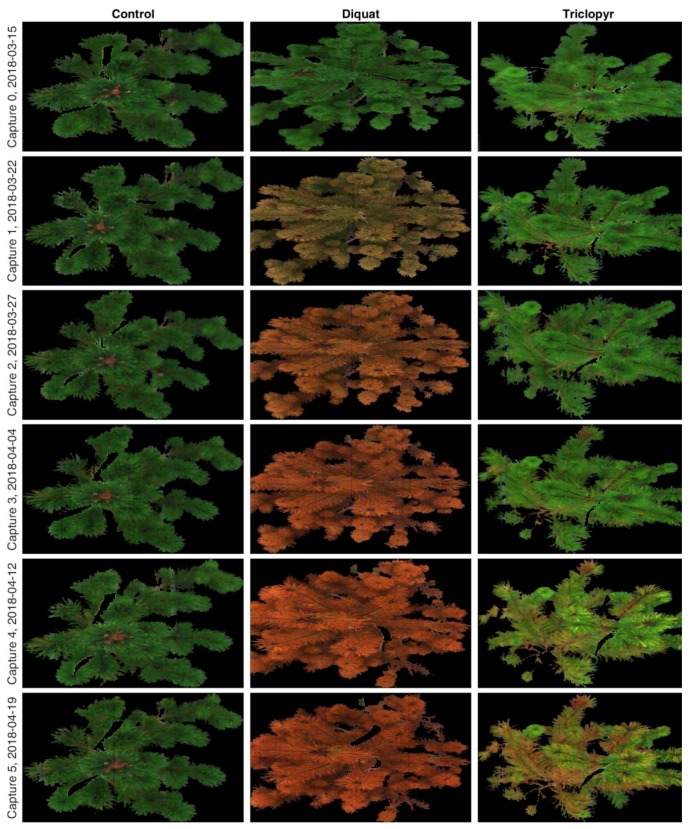
Time series imagery of an example tree from each treatment prior to treatment (Capture 0) and for the five weeks following treatment (Captures 1–5).

Images of good quality result from the preprocessing procedure described here. Some very slight striping is visible in the images [40], but no further steps were taken to compensate for this effect. Figure 5 shows the true color image time series for a selected tree from each treatment, which includes the control group, Diquat herbicide, and Triclopyr herbicide. The images show that Diquat very quickly turns the needles brown while Triclopyr works much more slowly. A detailed analysis of the herbicide effects is beyond the scope of this paper and will be the topic of another paper.

## 4. Conclusions

Close range imaging spectroscopy datasets of invasive conifers with and without herbicide treatment were recorded as a five-week time series. A white reference stick was included in every line of every image, but simple ratioing of vegetation pixels with white reference pixels did not lead to a satisfactory reflectance spectra. Instead, a correcting vector was calculated by ratioing a white reference measurement by pixels at the corresponding white reference columns identified in the original image. This approach may be helpful for comparable close-range spectroscopy applications and also for the radiometric correction of UAV-based imaging spectroscopy.

The resulting hyperspectral images will be used for analysis of the impact of herbicide treatments on the spectra of invasive conifers.

## Supplementary Materials

The following are available online at https://www.mdpi.com/1424-8220/19/7/1543/s1, Figure S1: True color depictions Capture 0, 2018-03-15; Figure S2: True color depictions Capture 1, 2018-03-22; Figure S3: True color depictions Capture 2, 2018-03-27; Figure S4: True color depictions Capture 3, 2018-04-04; Figure S5: True color depictions Capture 4, 2018-04-12; Figure S6: True color depictions Capture 5, 2018-04-19.
