# Supplementary material for: Preprocessing Ground-Based Visible/Near Infrared Imaging Spectroscopy Data Affected by Smile Effects
Source: Sensors (Basel). 2019 Mar 30;19(7):1543. doi: 10.3390/s19071543 (PMC6480459; doi:10.3390/s19071543)
Supplement: Supplementary file 1 [file sensors-19-01543-s001.pdf]

sensors

MDPI

*Supplementary Material*

# Preprocessing Ground-Based Visible/Near Infrared Imaging Spectroscopy Data Affected by Smile Effects

**Henning Buddenbaum [1,*], Michael S. Watt [2], Rebecca C. Scholten [1,3] and Joachim Hill [1]**

[1] Environmental Remote Sensing and Geoinformatics, Trier University, 54286 Trier, Germany; r.c.scholten@vu.nl (R.C.S.); hillj@uni-trier.de (J.H.)
[2] Scion, 10 Kyle Street, Christchurch 8011, New Zealand; Michael.Watt@scionresearch.com
[3] Faculty of Earth and Life Sciences, Vrije Universiteit Amsterdam, 1081 HV Amsterdam, the Netherlands;
* Correspondence: buddenbaum@uni-trier.de; Tel.: +49-651-201-4729

Received: 20 February 2019; Accepted: 26 March 2019; Published: 30 March 2019

This supplement contains true color depictions of all recorded trees (Figure S1–S6). The left column of each figure shows the control group, the middle column contains the group treated with Diquat herbicide, the right column contains the group treated with Triclopy herbicide.

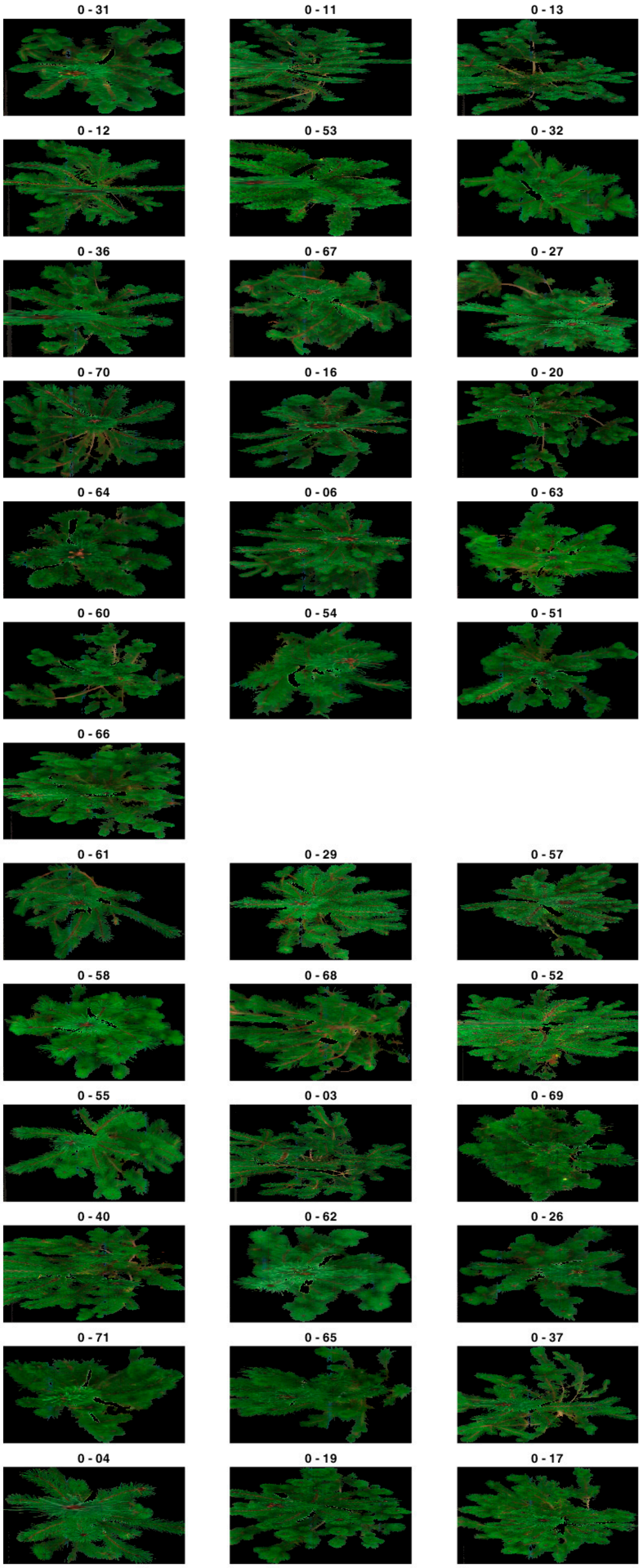


**Figure S1.** True color depictions Capture 0, 2018-03-15.

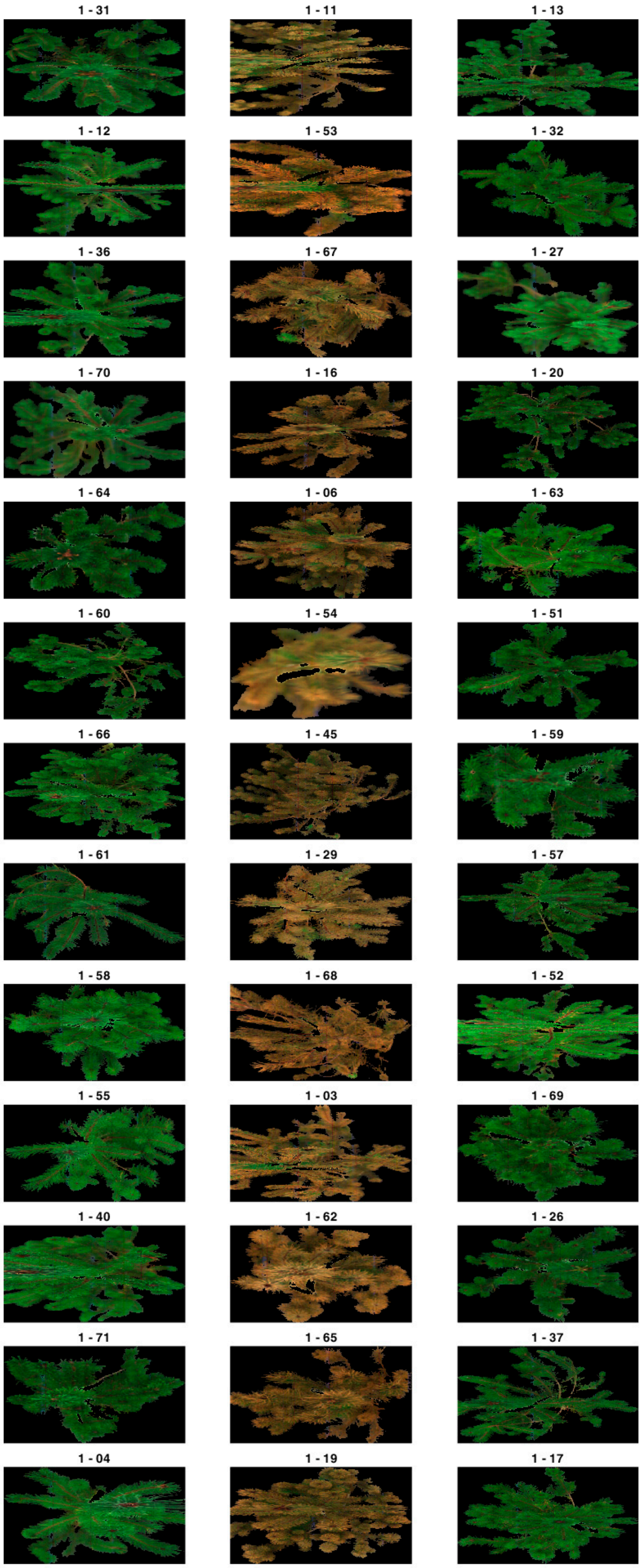


**Figure S2.** True color depictions Capture 1, 2018-03-22.

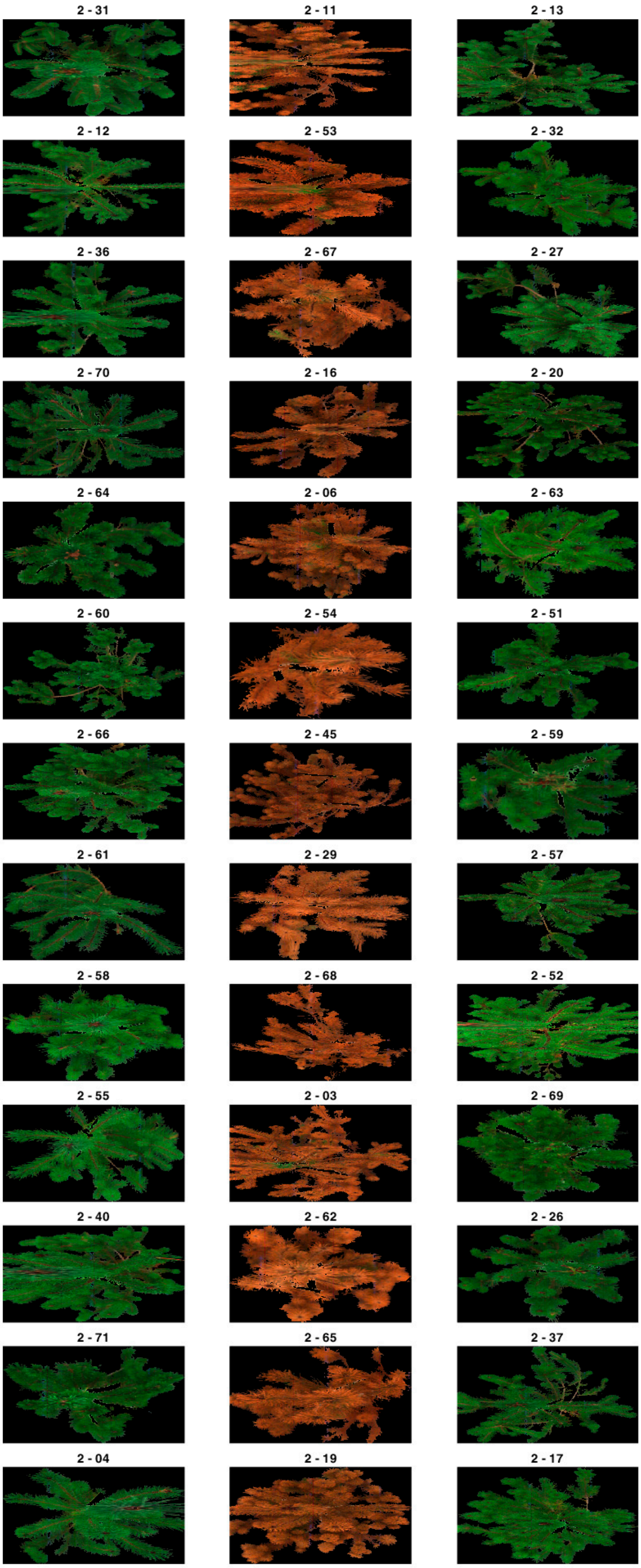


**Figure S3.** True color depictions Capture 2, 2018-03-27.

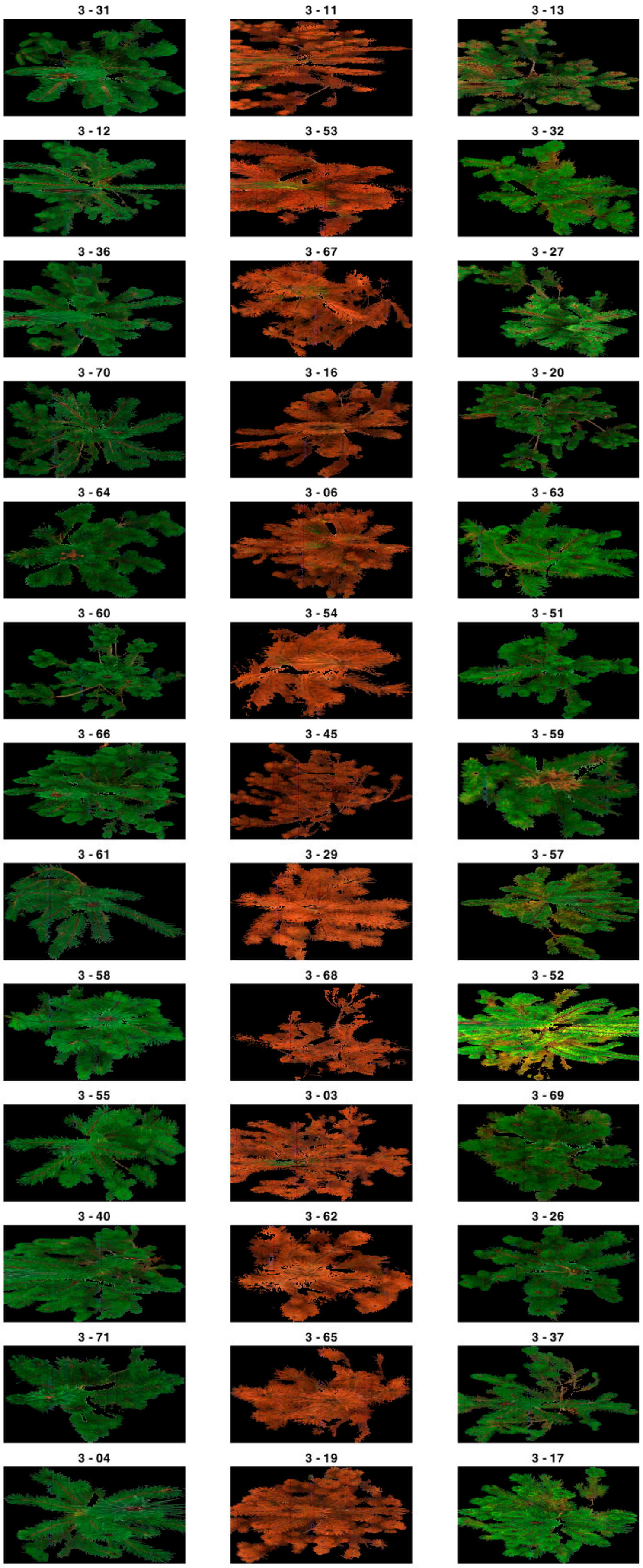


**Figure S4.** True color depictions Capture 3, 2018-04-04.

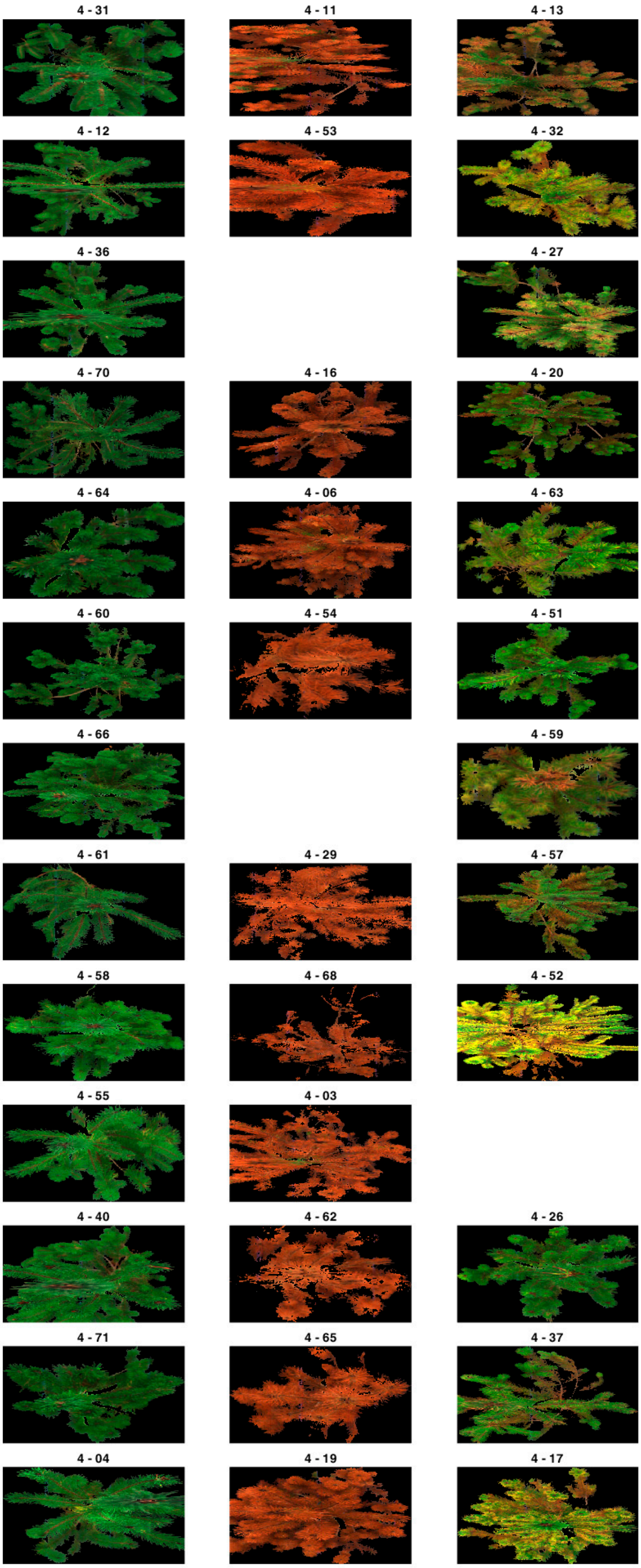


**Figure S5.** True color depictions Capture 4, 2018-04-12.

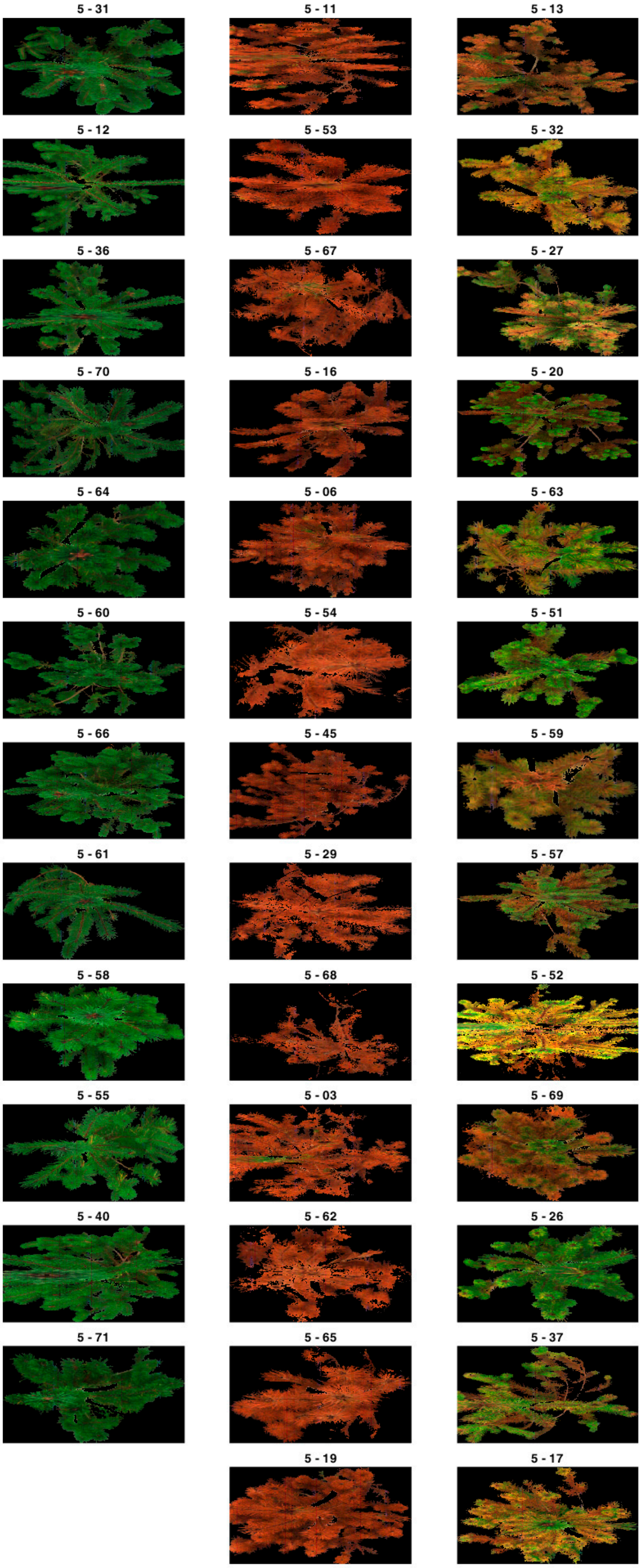


**Figure S6.** True color depictions Capture 5, 2018-04-19.
